# *Gene Composer* in a structural genomics environment

**DOI:** 10.1107/S1744309111027424

**Published:** 2011-08-13

**Authors:** Don Lorimer, Amy Raymond, Mark Mixon, Alex Burgin, Bart Staker, Lance Stewart

**Affiliations:** a Emerald BioStructures, 7869 NE Day Road West, Bainbridge Island, WA 98110, USA; b Seattle Structural Genomics Center for Infectious Disease, USA

**Keywords:** *Gene Composer*, structural genomics, *ClustalW*, protein structure, influenza, H1N1

## Abstract

The structural genomics effort at the Seattle Structural Genomics Center for Infectious Disease (SSGCID) requires the manipulation of large numbers of amino-acid sequences and the underlying DNA sequences which are to be cloned into expression vectors. To improve efficiency in high-throughput protein structure determination, a database software package, *Gene Composer*, has been developed which facilitates the information-rich design of protein constructs and their underlying gene sequences. With its modular workflow design and numerous graphical user interfaces, *Gene Composer* enables researchers to perform all common bioinformatics steps used in modern structure-guided protein engineering and synthetic gene engineering. An example of the structure determination of H1N1 RNA-dependent RNA polymerase PB2 subunit is given.

## Abstract 2

For structural biology applications, protein-construct engineering is guided by comparative sequence analysis and structural information, which allow the researcher to better define domain boundaries for terminal deletions and nonconserved regions for surface mutants. A database software application called *Gene Composer* has been developed to facilitate construct design.

## 1. Introduction

**Figure 1:**
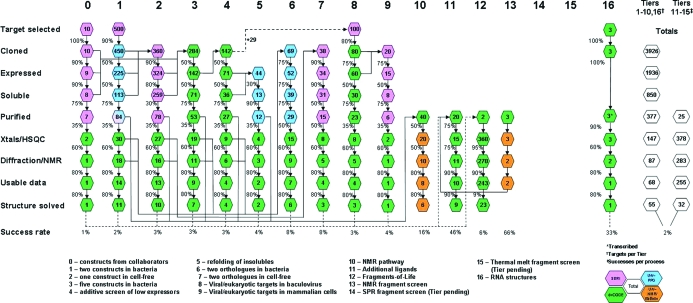
The SSGCID structure-determination pipeline. Tiers are represented as columns and described in the text at the bottom of the figure. Rows show the processes through which targets pass in each Tier. The numbers indicate how many targets are expected to pass through each step in the pipeline.

The Seattle Structural Genomics Center for Infectious Disease (SSGCID; http://www.ssgcid.org/home/index.asp) is devoted to the application of state-of-the-art structural genomics technologies to structurally characterize targeted proteins from NIAID Category A–­C pathogens and organisms. The goal is to create a collection of three-dimensional protein structures that are widely available to the broad scientific community and serve as a blueprint for structure-based drug development for infectious diseases. The SSGCID uses an escalating tier approach to protein production (Fig. 1 ▶). The overall SSGCID structure-determination pipeline involves a number of activities that are distributed between the Target Selection, Cloning & Expression Screening, Protein Production, Crystallization and Data Collection & Structure Solution teams. In order to maximize the likelihood of success of each target, yet minimize the cost per structure, we have adopted a multipronged serial escalation approach, whereby targets initially enter a standard high-throughput bacterial protein-expression system (Tier 1) and enter more resource-intensive ‘rescue pathways’ (Tiers 2–9) only after failing the initial approach. Tier 3 uses gene synthesis and multiple construct design as its technological focus.

To maximize our chances of success, we choose homologues and orthologues from multiple organisms with the goal of characterizing multiple examples of targets. It is well established in structural biology that making multiple different constructs of each target with various N- and C-terminal deletions or surface substitutions increases the chance of successfully obtaining soluble crystallizable protein (Chandonia & Brenner, 2006 ▶; Gräslund *et al.*, 2008 ▶). Deciding on where to make deletions and modifications can be accomplished in several ways. Making multiple sequence alignments using, for example, *ClustalW* (Chenna *et al.*, 2003 ▶; Larkin *et al.*, 2007 ▶) is straightforward and can help the researcher decide on conserved domains. Using secondary-structure information to identify structured domains can be accomplished by examining the structures of related entities using software tools such as *PyMOL* (DeLano, 2002 ▶) or *Coot* (Emsley & Cowtan, 2004 ▶). Creating constructs for cloning can be accomplished with molecular-biology tools such as Vector NTI (Invitrogen). Obtaining genomic DNAs from hard-to-obtain species or from metagenomic projects is difficult if not impossible. In such cases gene synthesis is the most attractive approach. We have developed a single computer application called *Gene Composer* that incorporates all of these functions into one package. The researcher can create information-rich multiple sequence alignments incorporating not only amino-acid sequences but also secondary-structural information from PDB files. Individual sequences can then be extracted from the alignment and used to create a fully codon- and sequence-engineered synthetic DNA sequence that can be incorporated into a virtual cloning strategy. Vector constructs are given unique vector-construct identifications (VCIDs) that are stored in the database. The VCID database can be queried and the necessary information extracted for each individual clone.

## 2. *Gene Composer* software

A typical design cycle starts by defining a desired target protein or family of targets. We first download the relevant target sequences from the SSGCID central target tracking database (CTTdb). We then pull in additional information from multiple sources such as FASTA files from GenBank (http://www.ncbi.nlm.nih.gov/), structure files from the Protein Data Bank (PDB; http://www.rcsb.org/pdb/home/home.do) or simple text (.txt) files with homologous sequences of related proteins or orthologs. *Gene Composer* automatically creates the familiar *ClustalW* (Chenna *et al.*, 2003 ▶; Larkin *et al.*, 2007 ▶) multiple sequence alignments, pointing out areas of conservation, gaps and dissimilar regions. Adding structural information is simple. Coordinate files from the PDB of related proteins or domains can be added to the alignment and used to display experimental information. Secondary-structural information is annotated and amino acids are identified that participate in ligand-binding sites, are water-exposed or form crystal contacts. At this point the researcher may decide that it is sufficient to express the activity-bearing domain only or that multiple amino-acid sequence variants be generated, including N- and C-terminal truncations, variants with surface mutations and single or combinations of tags at either end of the protein. Once domains and constructs have been identified, the user then defines the underlying DNA sequence, either as the native sequence or an engineered sequence. In the final step the user can virtually clone the constructs into vectors stored in the database using user-defined adapters and assemblies to create proteins with desired purification tags and features.

### 2.1. Alignment viewer and construct design

**Figure 2:**
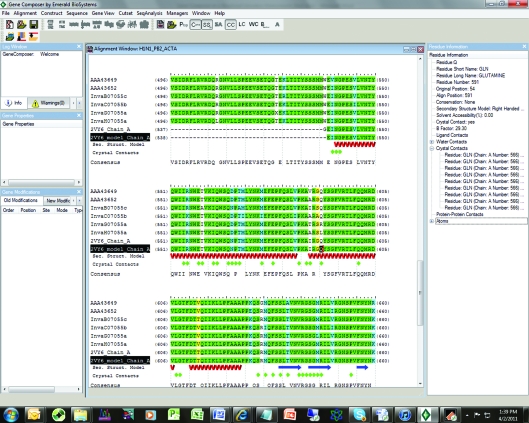
Alignment of influenza A RNA-dependent RNA polymerase PB2 subunit sequences from various influenza A strains with PDB entry 2vy6 (Guilligay *et al.*, 2008 ▶; Tarendeau *et al.*, 2008 ▶).

The H1N1 RNA-dependent RNA polymerase subunit PB2 offers an example of *Gene Composer* engineering leading to structure determination. This target has multiple SSGCID identifiers of the form Inva*X*.07055, where the *X* refers to multiple different strains or variants (Table 1 ▶). We started with several recent isolates and a published structure of the influenza A virus RNA-dependent RNA polymerase PB2 subunit (PDN entry 2vy6; Guilligay *et al.*, 2008 ▶; Tarendeau *et al.*, 2008 ▶) and created the alignment shown in Fig. 2 ▶. The degree of similarity is quite high amongst the SSGCID targets and 2vy6 (Guilligay *et al.*, 2008 ▶; Tarendeau *et al.*, 2008 ▶). The software not only extracts the structural information from the PDB but also displays (i) the chain sequence, which represents the protein put into crystallization trials, and (ii) the model, which represents the visible amino acids. In addition to displaying the conservation between sequences and the secondary structure, we can also interrogate the PDB file to extract other structure information such as *B* factors, crystal contacts, solvent accessibility and water contacts for any residue. For example, in Fig. 2 ▶ we have highlighted Gln591, which makes crystal contacts with Gln566, has a *B* factor of 29.3 Å^2^ and makes two water contacts in the structure.

**Figure 3:**
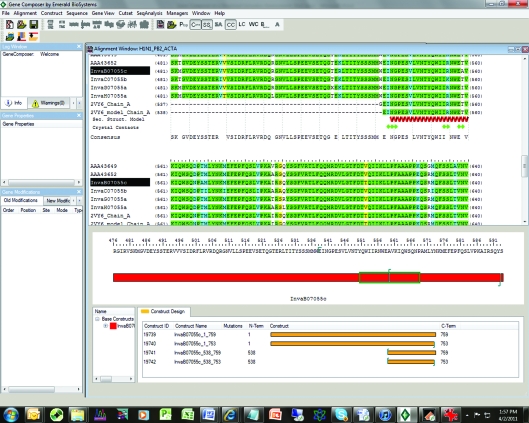
Construct design of InvaB.07055.c.

At this point the researcher can define a base construct from which to further design the expressed protein and to begin the gene-design process. Fig. 3 ▶ shows how N- and C-terminal truncations are set by inserting a character at the desired end points. *Gene Composer* then makes all combinations of truncations.

### 2.2. Construct Design Viewer

From information such as that displayed in Figs. 2 ▶ and 3 ▶ the user can now start to design constructs, making N- and C-terminal deletions, amino-acid insertions/deletions or surface mutations (Figs. 3 ▶, 4 ▶ and 5 ▶). We choose to make truncations at the N- and C-­termini or remove internally disordered loops by simply indicating in the Construct Design Viewer where the N- and C-termini should be by right-clicking at the position to start or stop (Fig. 3 ▶). To remove an internal sequence we simply highlight the residues to delete, right click and chose ‘delete residues’ from the list of options. Multiple combinations of truncations, mutations, insertion and deletions can be specified and all combinations are generated by the program.

### 2.3. Gene Design Module

**Figure 4:**
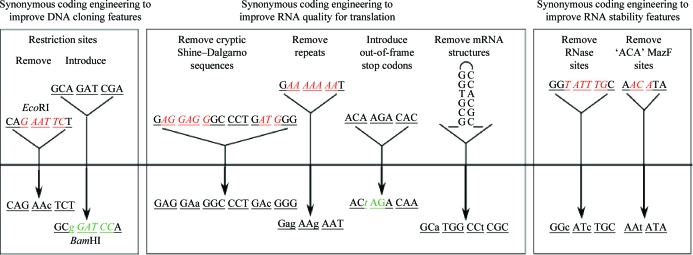
DNA-sequence modifications available to the user during the gene-design process (Lorimer *et al.*, 2009 ▶).

The features of the Gene Design Module have been presented in detail elsewhere (Lorimer *et al.*, 2009 ▶), so we will only briefly discuss them here. Once the user has defined the base construct to be made, he or she must now define a DNA sequence for use in the virtual cloning steps. The user can choose to use the natural sequence as found, for example, in GenBank or the PDB. Alternatively, the user may choose to design a gene from scratch by back-translating the target protein sequence using a codon-usage table (CUT) defined for the expression host. The CUT tables define the frequency of codon occurrence in the host genes, allowing the user to match the gene-codon frequencies to that of the host’s preferred codons and therefore avoid rare codons. Table 2 ▶ shows an example of the *Escherichia coli* codon-usage table. *Gene Composer* comes pre-loaded with CUTs for mammalian, insect cell/baculovirus, *E. coli* and combined *E. coli*/baculovirus genes. Once the basic back-translated gene has been designed, we can choose to introduce or remove restriction-enzyme sites, remove cryptic Shine–Dalgarno sequences, remove repeated sequences, introduce out-of-frame stop codons *etc.* (Fig. 4 ▶; Lorimer *et al.*, 2009 ▶).

**Figure 6:**
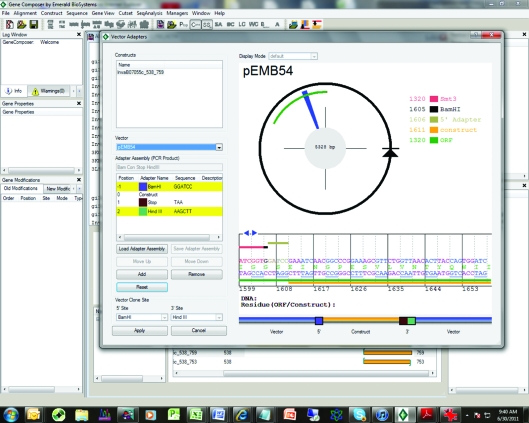
Virtual cloning in *Gene Composer*. Once contructs have been created as shown in Fig. 5 ▶ they can be virtually cloned. The vector is chosen from a pull-down menu and the appropriate adapters added to facilitate the cloning strategy. In this example a stop codon has been added to the end of all the constructs and *Bam*HI and *Hin*dIII adapters have been added.

Once all constructs have been decided on and the underlying DNA sequence has been defined the user can now perform a virtual cloning step in which the gene sequence is inserted, *in silico*, into a vector sequence (Fig. 6 ▶). The user can import vectors from commercial sources or create their own vectors. The user defines the potential cloning sites, vector-encoded tags, starts and stops. The user can also define adapters such as restriction-enzyme recognition sequences as well as tags and other types of features to be added to the target gene. These adapters are entered as DNA sequences and can be added to the ends of coding sequences as desired. These features are color-coded for easy visualization of the final construct-vector map. Adapters can be concatenated into assemblies that are stored for easy use. Once a gene sequence has been virtually cloned and stored in the database it is given a unique identifier called a Vector Construct ID or VCID. Once created, a VCID cannot be altered in any way so as to protect the integrity of the database. Deletion of VCIDs is password-protected to prevent accidental loss of data. Since the VCID is a software-generated piece of code, users can freely use it as a secure identifier in communication between parties.

## 3. Conclusions

**Figure 7:**
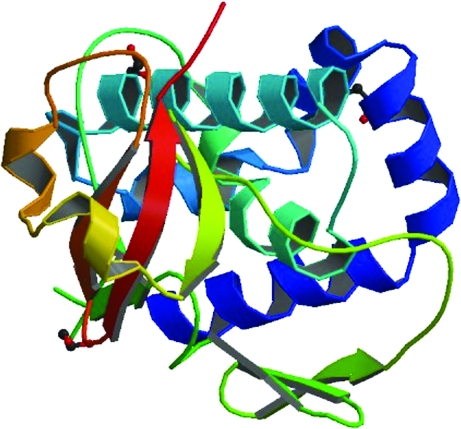
Structure model of influenza A RNA-dependent RNA polymerase subunit PB2 (PDB entry 3kc6; Yamada *et al.*, 2010 ▶). The expressed construct was designed using *Gene Composer* as described in this paper. The expression and purification methods are as described in Raymond *et al.* (2011 ▶). The figure was downloaded from the PDB.

Structural genomics efforts are becoming commonplace in the field of functional and structural biology. For example, there are currently 36 centers tracked in the Protein Data Bank, including sites in the US, Japan and Europe. As of July 2011 these centers have submitted 10 370 structures to the PDB. At present the SSGCID is the eighth most productive center tracked. A key to our success is the ability to clone, express, purify and crystallize multiple versions of any single target in our pipeline. The SSGCID uses an escalating-tier approach to protein production (Fig. 1 ▶). Tier 3 uses gene synthesis and multiple construct design as its technology. As such, in Tier 3 *Gene Composer* provides an excellent tool for performing rational construct design using parameters such as sequence homology, synthetic DNA engineering and structural features. Examples of Tier 3 rescue are discussed in Raymond *et al.* (2011 ▶). For the example given here of the H1N1 RNA-dependent RNA polymerase subunit PB2, specific DNAs were difficult if not impossible to obtain for this work and therefore gene synthesis was the only option. Multiple versions of the protein were created and expression and purification were accomplished (Raymond *et al.*, 2011 ▶; Yamada *et al.*, 2010 ▶). Purified protein entered into crystallization trials and a structure model was determined (Fig. 7 ▶). The structure and its impact on our understanding of cross-species transmission are discussed elsewhere (Yamada *et al.*, 2010 ▶).

## 4. Availability and requirements

*Gene Composer* software can be downloaded for free from http://www.genecomposer.net by following a simple click-through registration license. The operating system required is Windows 2000/XP and the hardware is a Pentium 4 or Athlon at 1 Ghz with 1 GB RAM. *Gene Composer* is a registered trademark of Emerald BioStructures Inc.

## Figures and Tables

**Figure 5:**
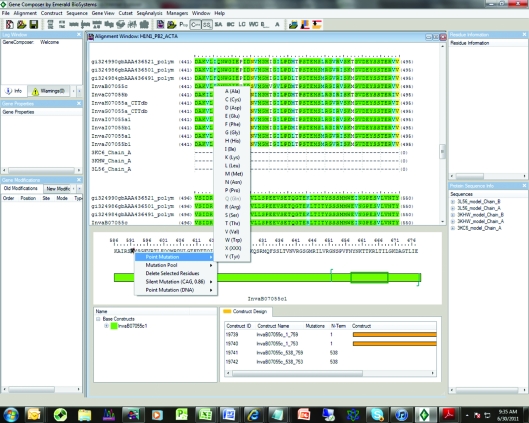
Construct Design Viewer. In the lower panel the user can make N- or C-terminal deletions, amino-acid insertions, deletions or surface mutations.

**Table 1:** PB2 homologues from various species The SSGCID targets are named Inva*X*.07055.a, where *X* = B (A/Yokohama), C (A/equine), G (A/H1N1) or H (A/chicken). The model 2vy6 is for A/California from an isolate from 1978 (Guilligay *et al.*, 2008 ▶; Tarendeau *et al.*, 2008 ▶).

SSGCID name	Organism ID	Reference
InvaB.07055.a	A/Yokohama/2017/03 (H3N2)	http://www.ncbi.nlm.nih.gov/genomes/FLU/
InvaC.07055.a	A/equine/Prague/1/1956 (H7N7)	http://www.ncbi.nlm.nih.gov/genomes/FLU/
InvaG.07055.a	A/H1N1 subtype	http://www.ncbi.nlm.nih.gov/genomes/FLU/
InvaH.07055.a	A/chicken/Nanchang/3-120/2001 (H3N2)	http://www.ncbi.nlm.nih.gov/genomes/FLU/
2vy6	A/California/10/1978 (H1N1)	Tarendeau *et al.* (2008 ▶)

**Table 2:** *E. coli* codon-usage table (CUT) The indicated values represent the fractional occurrence of each codon in the *E. coli* transcriptome. Data from Kazusa (http://www.kazusa.or.jp/codon/).

Codon	Amino acid	Frequency
TTT	Phe	0.21
TTC	Phe	0.79
TTA	Leu	0.03
TTG	Leu	0.02
TCT	Ser	0.39
TCC	Ser	0.39
TCA	Ser	0.02
TCG	Ser	0.04
TAT	Tyr	0.20
TAC	Tyr	0.80
TAA	X	0.83
TAG	X	0.17
TGT	Cys	0.20
TGT	Cys	0.80
TGA	X	0.00
TGG	Trp	1.00
CTT	Leu	0.05
CTC	Leu	0.06
CTA	Leu	0.01
CTG	Leu	0.83
CCT	Pro	0.08
CCC	Pro	0.01
CCA	Pro	0.08
CCG	Pro	0.82
CAT	His	0.17
CAC	His	0.83
CAA	Gln	0.14
CAG	Gln	0.86
CGT	Arg	0.73
CGC	Arg	0.24
CGA	Arg	0.00
CGG	Arg	0.01
ATT	Ile	0.12
ATC	Ile	0.86
ATA	Ile	0.02
ATG	Met	1.00
ACT	Thr	0.36
ACC	Thr	0.56
ACA	Thr	0.02
ACG	Thr	0.05
AAT	Asn	0.09
AAC	Asn	0.91
AAA	Lys	0.81
AAG	Lys	0.19
AGT	Ser	0.01
AGC	Ser	0.15
AGA	Arg	0.02
AGG	Arg	0.00
GTT	Val	0.57
GTC	Val	0.07
GTA	Val	0.21
GTG	Val	0.15
GCT	Ala	0.45
GCC	Ala	0.07
GCA	Ala	0.28
GCG	Ala	0.21
GAT	Asp	0.28
GAC	Asp	0.72
GAA	Glu	0.83
GAG	Glu	0.17
GGT	Gly	0.48
GGC	Gly	0.50
GGA	Gly	0.00
GGG	Gly	0.01
